# A new perspective on HIV: effects of HIV on brain-heart axis

**DOI:** 10.3389/fcvm.2023.1226782

**Published:** 2023-08-04

**Authors:** Honghua Shao, Sijun Li

**Affiliations:** Department of Internal Medicine, The Fourth People's Hospital of Nanning, Nanning, China

**Keywords:** human immunodeficiency virus, central nervous system, cardiovascular system, brain, heart

## Abstract

The human immunodeficiency virus (HIV) infection can cause damage to multiple systems within the body, and the interaction among these various organ systems means that pathological changes in one system can have repercussions on the functions of other systems. However, the current focus of treatment and research on HIV predominantly centers around individual systems without considering the comprehensive relationship among them. The central nervous system (CNS) and cardiovascular system play crucial roles in supporting human life, and their functions are closely intertwined. In this review, we examine the effects of HIV on the CNS, the resulting impact on the cardiovascular system, and the direct damage caused by HIV to the cardiovascular system to provide new perspectives on HIV treatment.

## 1. Introduction

Acquired immune deficiency syndrome (AIDS) is a condition characterized by systemic immune deficiency caused by infection with the human immunodeficiency virus (HIV). HIV could evade the innate immune detection in the eclipse phase that follows virus inoculation and establish a foothold in host cells, which lead to massive virus propagation in T cells and significant T cell death (1). HIV primarily targets cells that possess CD4 receptors, particularly CD4+ T cells, leading to immune system dysfunction, damage to multiple systems within the body, and severe consequences (2–6). Each year, an estimated 2.5 million individuals become infected with HIV, and HIV-related illnesses contribute to around 2.1 million deaths (7). Towards the end of the previous century, highly active antiretroviral therapy (ART) was introduced in clinical practice due to its effectiveness in suppressing the viral load of HIV throughout the body, reducing mortality rates, and lowering the incidence of opportunistic infections in individuals with AIDS (8). Consequently, AIDS transformed from a fatal disease to a chronic condition, with the life expectancy of people living with HIV becoming comparable to that of the general population, primarily due to the widespread use of ART (9). However, it's important to note that HIV can impact multiple systems, in addition to the immune system (10–15), such as the central nervous system (CNS), cardiovascular system (CS), respiratory system, digestive system, urinary system, and endocrine system. Compared to individuals without HIV, those infected with HIV have a higher risk of developing neurological and cardiovascular diseases, which tend to occur at an earlier stage, significantly impacting their quality of life (9). The interconnectedness of various organ systems implies that pathological changes in one system can affect the functioning of other systems (16). As a result, even if HIV primarily affects a specific system, its interaction with other systems can disrupt their normal physiological functions, exacerbating the disease in patients. Despite this complexity, most HIV treatment and research remain focused on individual systems, often overlooking the comprehensive interplay among different systems.

The regulatory mechanism of the CNS plays a significant role in the functioning of CS (11). A complex interaction exists between the CNS and CS, where the brain acts as the higher regulatory center, and the autonomic nervous system (ANS) is the final effector in regulating cardiovascular activities. Various brain regions, including the prefrontal cortex (PFC), insular cortex, amygdaloid nucleus, cingulate cortex, hypothalamus and brainstem, form intricate networks within the nervous system to regulate the ANS. Ultimately, the ANS controls CS, establishing the brain-heart axis (17, 18). In this review, our primary focus is to discuss the effects of HIV on the CNS and its subsequent impact on the CS. We also discuss the direct damage caused by HIV to the CS itself, and it is important to note that our discussion specifically centers on the damage caused by HIV and does not cover opportunistic infections resulting from HIV. The references were obtained from PubMed (https://pubmed.ncbi.nlm.nih.gov/). The main search keywords were “HIV”, “AIDS”, “brain”, “central nervous system”, “heart”, “cardiovascular”, “microglia”, “astrocyte”, “neuron”, “prefrontal cortex”, “insular cortex”, “amygdaloid nucleus”, “cingulate” “cortex”, “hypothalamus”, “brainstem”, “heart muscle”, “endocardium”, “pericardium”, “artery” and “Arrhythmia” and so on. The years of search was from 1949 to 2023.

## 2. Effects of HIV on CNS

The entry of HIV into a target host cell involves the interaction between the viral envelope glycoprotein gp160 and the host receptor. The gp160 is transported through the cellular secretory pathway to the plasma membrane (19). During this process, it undergoes extensive glycosylation, oligomerization into trimers, and proteolytic maturation mediated by a cellular furintype protease that cleaves it into the mature gp120 and gp41 Env subunits, which interact with the CD4 receptor, CC chemokine receptor 5 (CCR5), or C-X-C chemokine receptor 4 (CXCR4) on the surface of the host cell (19, 20). Consequently, HIV has a propensity to invade cells that express CD4 receptors on their cell membranes, including microglia, T lymphocytes, mononuclear/macrophages, and dendritic cells (2–5). Although ART can effectively reduce viral load and the occurrence of opportunistic infections, it is unable to completely eradicate the dormant virus within the host. HIV is known as a neurotropic virus that can cause damage to the nervous system (21). Therefore, to properly diagnose and treat HIV-related neurological damage, it is crucial to understand the relationship between HIV and various cells within the CNS, identify the susceptible sites of the CNS, and comprehend the mechanisms by which HIV induces damage to the CNS.

### 2.1. Cells in CNS Infected by HIV

In peripheral system, the human leukocyte antigen (HLA) and cytotoxic T lymphocyte (CTL) play the key roles in cell-mediated adaptive immune response (22, 23). The primary function of HLA is to present endogenous and exogenous antigens to T lymphocytes for recognition and response (24). The HLA molecules are cell surface glycoproteins, including two main classes: HLA class I and HLA class II molecules. HLA class I molecules are predominantly involved in the immune defense of intracellular pathogens, and HLA class II play an essential role in displaying peptides from extracellular pathogens (25). Lazaryan et.al suggested that a higher frequency of HLA genotypic supertypes correlated with a higher mean viral load and lower mean CD4 count (26).The CTL delivers a cocktail of cytotoxic substances from secretory lysosomes (cytolytic granules) to destroy the target (23). Madrid-Elena et.al considered that CTLs are suppressed after HIV infection, which may be related to miRNA((hsa-miR-10a-5p) (27). However, the effects of HIV on CNS various cells are more complex.

**Figure 1:**
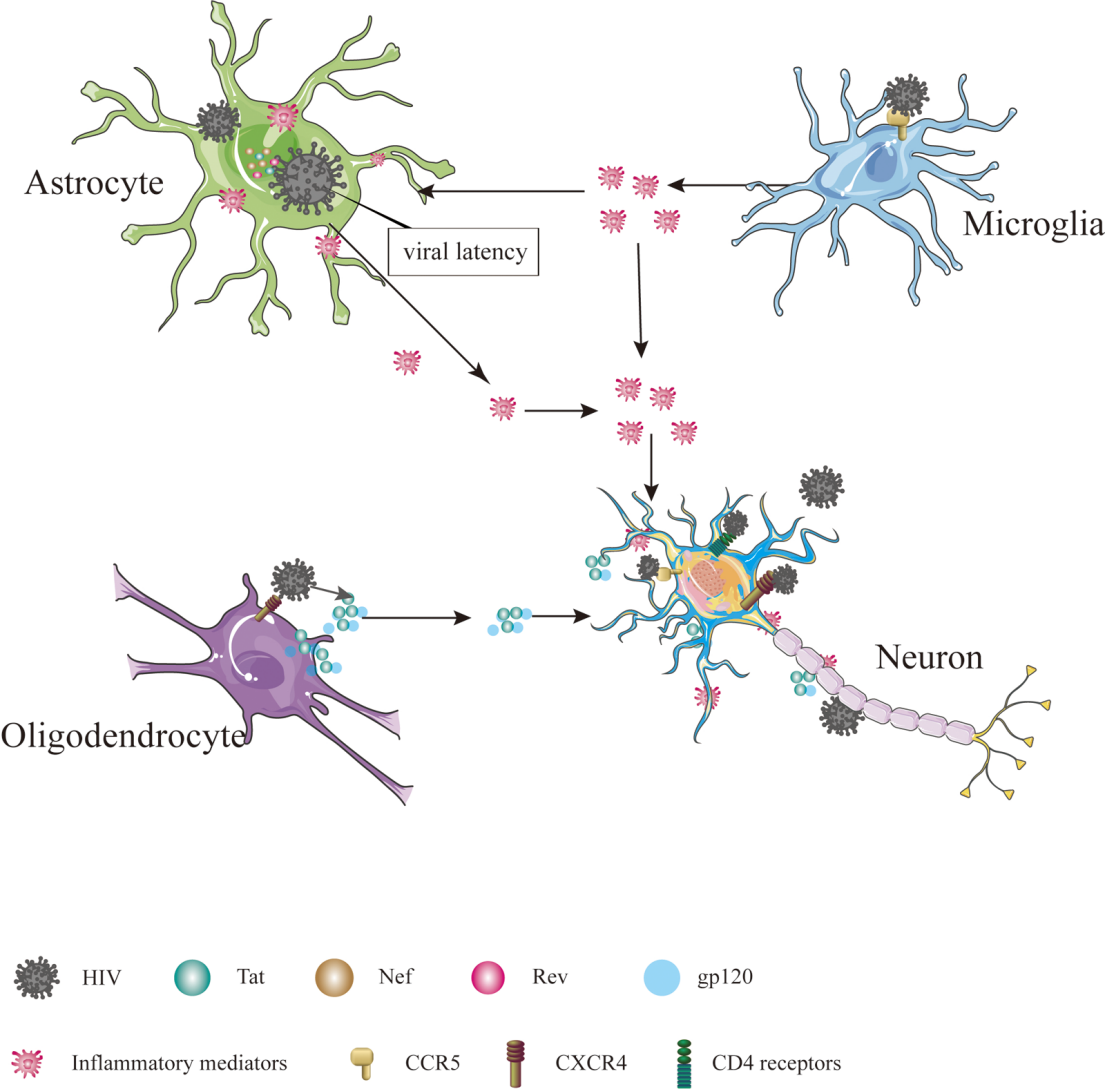
Cells in CNS infected by HIV. The cells in CNS, including astrocyte, microglial, oligodendrocyte and neuron, are affected directly or indirectly by HIV, ultimately leading to dysfunction, injury and death.

The precise mechanism by which HIV enters the nervous system is not yet fully understood, but several hypotheses have been put forward. One such hypothesis is the “Trojan horse” theory, suggesting that the virus gains access to the CNS using monocytes or infected CD4+ T lymphocytes as a means of transport (6). Another hypothesis, proposed by Ruifen Xu et al., suggests that HIV can disrupt the integrity of the blood-brain barrier (BBB) by affecting the tight connections between endothelial cells that form the blood vessels, thereby allowing entry into the brain (28). Banks et al. proposed that HIV crosses the BBB through cellular transfer facilitated by the viral envelope protein gp120 (29). Additionally, Kamerman suggested that HIV may directly infect peripheral nerve fibers and subsequently travel in a retrograde manner to reach the CNS (30). Once inside the CNS, HIV invades various cell types, including astrocytes, microglia, oligodendrocytes and neurons Table 1 and Figure 1 (31).

**Table 1:** Cells in CNS infected by HIV.

Cell-type	Physiological function	The way HIV damages cells	Pathological features caused by HIV
Astrocyte	Provide vital energy support to neurons (39, 97)	1. Endocytosis and maintain viral latency (33–35) 2. Production and release of viral proteins (36)	1. Energy metabolism disorders (38–40) 2. Formation of glial scars (41, 42) 3. Influence the BBB (43)
Microglia	Innate immune (44)	Direct HIV infection (48)	Release inflammatory chemokines and cytokines (49, 50)
Oligodendrocytes	Form the myelin sheath of the axon (51)	Production and release of viral proteins (55)	1. Inhibition of cell growth, developmental retardation,cell death (56, 57) 2. WM damage (52, 53)
Neuron	Fundamental functional units (58)	1. Direct HIV infection (59–61) 2. Viral proteins disrupt the Ca^2+^ homeostasis (62)	1. E/I imbalance (65) 2. Neuronal dysfunction, injury and death (62)

#### 2.1.1. Astrocyte

Interestingly, HIV infectious virions and viral proteins have been detected in astrocytes, despite these cells lacking CD4 receptors and CCR5 (32). Astrocytes can internalize HIV through endocytosis and maintain viral latency by suppressing viral replication (33–35). Although HIV cannot replicate, it can interact with astrocytes, leading to the production and release of viral proteins such as Tat, Nef and Rev, contributing to inflammation and nerve damage (36). Inflammation in the nervous system can disrupt the energy metabolism of astrocytes (37). Given that astrocytes provide vital energy support to neurons, any disturbance in their energy metabolism can lead to neuronal energy metabolism disorders (38–40). In addition, inflammation can stimulate the proliferation and differentiation of astrocytes, resulting in the formation of glial scars, which impede axon regeneration and cell migration and directly affect neuronal growth (41, 42). More importantly, as a significant component of BBB, astrocytes may influence the function and structure of the BBB when affected by HIV (43).

#### 2.1.2. Microglia

Microglia, a component of the innate immune system, are often referred to as the “macrophages” of the CNS (44). Excessive activation of microglia can contribute to the development of various neurological disorders, including Alzheimer's disease (45), Parkinson's disease (46) and HIV-associated neurocognitive disorder (HAND) (47). Microglia more susceptible to HIV infection due to CCR5 is expressed on the cell surface (48). Prolonged microglia activation can increase inflammatory chemokines and cytokines, adversely affecting astrocytes and neurons and increasing neurotoxic substances that induce neuronal apoptosis, ultimately contributing to the development of HAND (49, 50).

#### 2.1.3. Oligodendrocytes

Oligodendrocytes, which form the myelin sheath of the axon of the brain, do not touch the axon directly but repeatedly wrap the extension of axons and then gather the myelin sheath of multiple helical structures (51). The formation of the myelin sheath increases the speed and energy efficiency of nerve conduction by facilitating the jump in signal transmission (52), which is essential for cognitive functions (53). The involvement of HIV infection in oligodendrocytes, which specifically express the CXCR4 receptor, remains a topic of debate (54). However, research conducted by Zou et al. indicates that damage to oligodendrocytes is caused by the release of the Tat protein by HIV, resulting in the inhibition of cell growth, developmental retardation, and even cell death (55). In addition to Tat, the gp120 protein has been implicated in oligodendrocyte injury, as it inhibits myelination, induces dysfunction and triggers apoptosis of oligodendrocytes (56, 57).

#### 2.1.4. Neuron

Neurons are the fundamental functional units of the nervous system and are considered non-regenerative cells (58). The ability of HIV to infect neurons is still a matter of debate. Previous studies have shown that adult neurons express CXCR4, CCR5 and CCR3 on their surface and that CD4 receptors may also be present in certain neurons located in the cerebellum, thalamus, pons and hippocampus (59–61). Nath et al. proposed that proteins such as Tat and gp120 can disrupt the Ca^2+^ homeostasis of neurons and normal neuronal functions, leading to neuronal dysfunction, injury and death (62). Exposure of neurons to Tat and gp120 has been shown to increase the levels of glutamate (Glu) and the expression of N-methyl-D-aspartic acid (NMDA) receptors (63, 64). Furthermore, gp120 can enhance the inhibitory function of neurons by increasing the expression of gamma-aminobutyric acid (GABA) type A receptors (65).

### 2.2. The areas of CNS vulnerable to HIV

HIV can have wide-ranging effects on the metabolism, neural network structure, and volume of the brain (66–68). Certain cortical and subcortical regions of the brain, including the medial prefrontal cortex (MPFC), basal ganglia, and hippocampus, which play crucial roles in cognition and emotion, are susceptible to the impact of HIV and HIV-related proteins, leading to functional and structural alterations within these regions (69, 70). Jason J. Paris et al. has shown that the Tat protein can induce microglia activation in the MPFC, anterior cingulate cortex (ACC), amygdala, nucleus accumbens, and dentate gyrus, suggesting that these regions are also vulnerable to the effects of HIV (71). Furthermore, Sara R. Nass et al. demonstrated that HIV Tat protein not only affects the PFC and amygdala but also reduces oxytocin levels in the hypothalamus (72). Theresa K. Smit et al. dissected the brains of HIV-infected individuals and identified the presence of HIV in the frontal, parietal, and occipital lobes (73). Jing Sui et al. utilized head MRI scans and observed abnormal changes in gray matter volume in the thalamus, prefrontal lobe, precuneus, posterior parietal lobe, and occipital lobe of HIV-infected patients (68). Additionally, Eleni Giatsou et al. demonstrated continuous HIV replication in multiple brain regions, including the cerebellum, bulbar region, temporal lobe, substantia nigra, and caudate nucleus (74).

### 2.3. HIV-induced CNS damage

The acute HIV infection period is brief and peak viremia predicts a viral set point that occurs 4–5 weeks following infection (75). Following the detection of HIV RNA, highly activated CD8+ T cells expand and peak approximately 2 weeks following peak viral load (76). Early in HIV infection, the count of CD4+ T cells is greater than 500 cells/ml (77). Days 8 to 30 after infection are characterized by massive viral replication and the death of large numbers of CD4+ T cells (78). Bolzenius et al. indicated that brain volume alterations may occur in acute infection, with the most prominent differences evident in the later stages of acute HIV infection (79). Nevertheless, Hellmuth et.al suggested that no structural neuroimaging abnormalities were observed in acute HIV infection (80). With the success of ART, HIV-seropositive patients can now live for many years despite chronic viral infection. The CD4+ count of chronic HIV-infected patients receiving ART remain above 250 cells/ml (81). Chronic HIV infection causes persistent low-grade inflammation that induces premature aging of the immune system including senescence of memory and effector CD8+ T cells. CD8+ T cell dysfunctions associated with chronic HIV infection may lead to chronic disturbances in the ability of these cells to properly engage with infected target cells (82). In the chronic phase of HIV infection, the HIV-related damage to the CNS has shifted towards chronic lesions, leading to conditions such as cognitive impairment, behavioral disorders, and motor dysfunction collectively known as HAND (83). However, the diagnosis and treatment of HIV-related HAND face significant challenges because the incidence of HAND does not show a significant correlation with the concentration of HIV-RNA (viral load) or the count of CD4 cells, which are commonly used markers to monitor HIV infection. Instead, the risk of developing HAND tends to increase as the duration of HIV infection progresses (84–87). Previous studies have identified two stages of HIV-induced CNS damage. The first stage involves metabolic disorders (88–90), while the second involves structural lesions (91).

#### 2.3.1. Metabolic dysfunction of CNS

**Figure 2:**
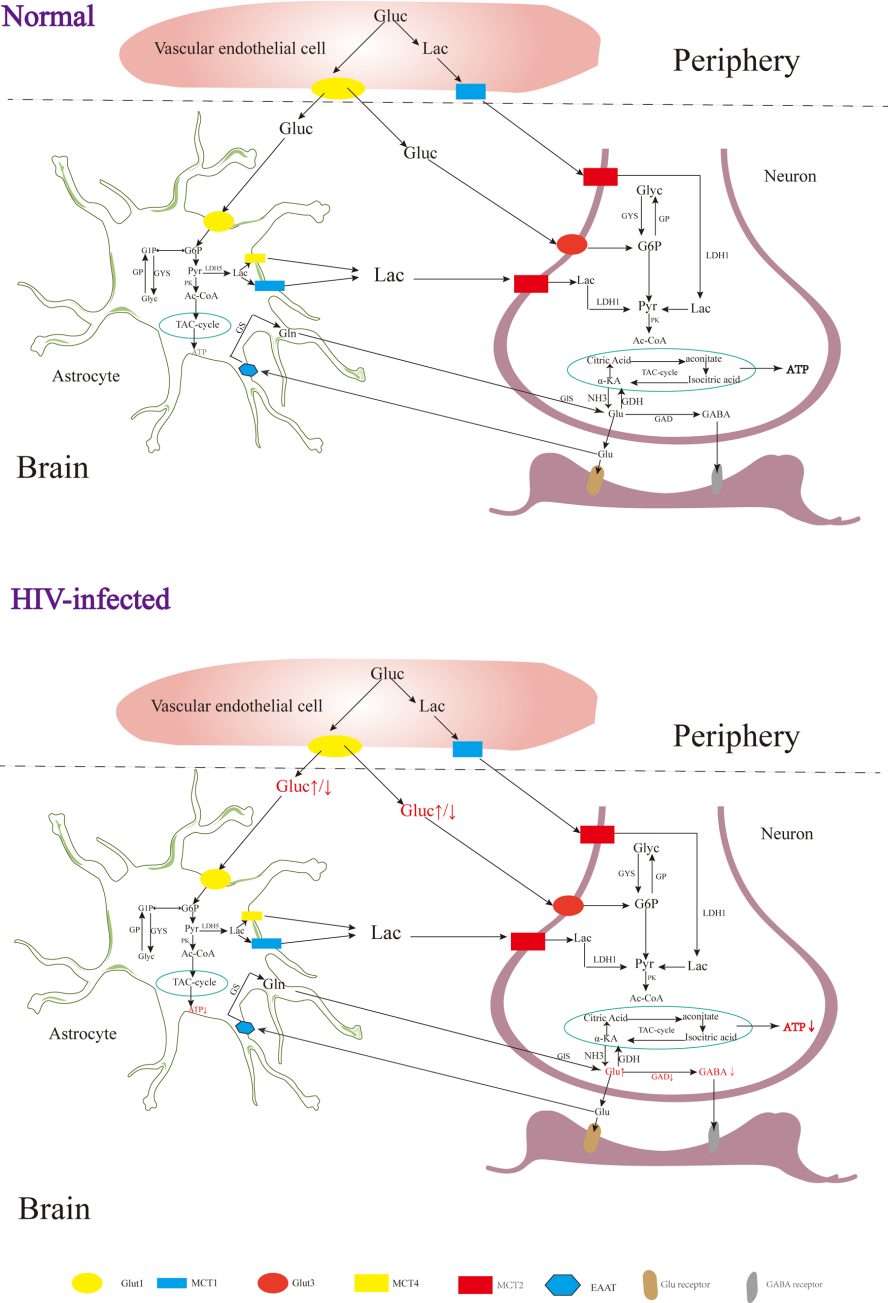
The metabolism of the nervous system. Gluc, glucose; G6P, glucose 6 phosphate; G1P, glucose 1 phosphate; GP, glycogen; Pyr, pyruvate; Lac, lactate; Ac-COA, acetyl coenzyme A; α-KA, α-ketoglutaric acid; Suc-CoA, succinic acid coenzyme A; Suc, succinic acid; Suc-sem, succinic acid semialdehyde; GYS, glycogen synthase; GP, glycogen phosphorylase; PK, pyruvate kinase; LDH, lactate dehydrogenase; GDH, glutamate dehydrogenase; GDA, glutamic acid decarboxylase; GS, glutamine synthetase; Gls, glutaminase; GABA-T, GABA transaminase; SSADH, succinic semialdehyde; Glut, glucose transporter; MCT, monocarboxylic acid transporter; EAAT, excitatory amino acid transporter.

In the early stages of HAND, patients may experience mild difficulties with concentration, motor symptoms, and focal cortical deficits such as apraxia, agnosia, or aphasia (92), although there is no significant observable loss of neurons or other substantial pathological changes detected in HIV patients during this stage (93). In addition, although neuroimaging studies also do not typically reveal significant lesions or structural changes, alterations in certain metabolites have been observed Figure 2 (94). The metabolism of the nervous system is a complex physiological process involving the production of sufficient ATP for energy and the synthesis of neurotransmitters essential for maintaining normal physiological brain functions (95). Upon entering the nervous system, glucose in the blood undergoes a series of enzymatic reactions catalyzed by key enzymes like hexokinase, 1,6-diphosphofructokinase-1 and pyruvate kinase, which can convert glucose into pyruvic acid (Pyr), and subsequently, the tricarboxylic acid cycle generates ATP, providing energy for neuronal functions (96). Neuron-astrocyte interaction is another important mechanism for energy supply in the brain (39, 97). Glucose in astrocytes is converted into glycogen under the influence of glycogen synthase (GYS), which is then gradually broken down into pyruvate (98). Some of these pyruvates are converted into lactate by the enzyme lactate dehydrogenase 5 (LDH5), which is then transported to neurons and converted back into pyruvate by lactate dehydrogenase 1 (LDH1), entering the tricarboxylic acid cycle pathway (99). During this cycle, α-ketoglutaric acid in neurons can react with NH3 to form Glu (100, 101), which can be further converted into gamma-aminobutyric acid (GABA) through the catalytic action of glutamic acid decarboxylase (GAD) (102, 103).

Rottenberg D et al. observed glucose hypermetabolism in the thalamus and basal ganglia, along with hypometabolism in the cortex and subcortical gray matter during the early stages of HIV-associated HAND (104). Zeping Wang et al. found that after receiving ART, glucose metabolism in the frontal cortex of HIV-infected individuals was enhanced, suggesting that the damage to the frontal cortex at this stage is potentially reversible (105). After HIV infection, the level of ATP in the brain is decreased, and the level of ATP in the peripheral blood is increased (106, 107). Tat, an HIV protein, has been implicated in increasing Glu levels and decreasing GABA levels in the brain (108), which may be related to the inhibition of GAD by HIV (109). In the early stages of HIV infection, increased levels of Glu in the brain can contribute to excitatory neurotoxicity, leading to clinical symptoms such as reduced attention (110, 111). As the disease progresses, individuals with HIV and cognitive deficits tend to have lower Glu levels in the parietal gray matter, indicating that decreased Glu levels may contribute to cognitive impairment (112). In addition to HAND, HIV-associated epilepsy is also a significant challenge, with reported incidence rates ranging from 6.1% to 83.75% (113, 114). Although the underlying mechanisms of HIV-related epilepsy are not yet fully understood, it is believed to involve an imbalance of Glu and GABA (108, 115).

#### 2.3.2. Structural damage of CNS

As the disease advances, HIV patients often experience worsening motor dysfunction and cognitive impairments, which become increasingly noticeable and drastically hinder their daily activities. Completing complex tasks often takes them longer, and they may sometimes become unable to accomplish them adequately. Specific motor dysfunctions may include slower, less precise movements, clumsiness, an unstable gait, and a loss of balance (86). Simioni et al. discovered that these patients exhibited significant signs of frontal lobe release, cramps and hyper-reflexes, particularly in the legs (86). At this stage, neuroimaging studies have identified white matter signal abnormalities as the most prevalent (116, 117). White matter (WM) consists of the aggregation of nerve fibers in the central nervous system, predominantly composed of axons and oligodendrocytes of neurons, making it a vital component of the neural network (118). The release of Tat protein and gp120 by HIV can result in the injury and apoptosis of oligodendrocytes (55–57). Consequently, the damage and apoptosis of oligodendrocytes impair the formation of effective myelin sheaths, leading to reduced nerve conduction efficiency and cognitive dysfunction in the brain (52, 53). HIV can also have an impact on the axons of neurons, leading to abnormal neural network connections (119). In advanced cases of HAND with severe cognitive dysfunction and motor symptoms, there may be a connection to white matter injury, whereby white matter injury can disrupt the limbic circuit and damage cortico-cortical connections, thereby affecting intelligence and cognitive function (120). The integrity of nerve fiber connections in various regions such as the corpus callosum, cingulate gyrus, anterior limb of the internal capsule, cerebral foot, anterior crown, and frontal-occipital lobe tract is closely related to balance and gait function (121). HIV-induced damage to the brain's white matter can interfere with nerve fiber connections, leading to abnormal gait in patients with HAND (121). Falls have been associated with injuries to the total white matter, periventricular white matter, and deep white matter (122). Blahak et al. suggested that white matter injury can disrupt the prefrontal subcortical motor circuit, resulting in impaired balance and an increased risk of falls (123). In addition to white matter injury, advanced HAND patients may exhibit decreased subcortical volume, caudate nucleus volume, cerebral malacia foci, and brain atrophy (66, 124, 125). HIV can also contribute to other acute central nervous system diseases, such as stroke. Bertrand et al. revealed a higher incidence of stroke in HIV patients compared to the same age group (126). While men generally have a higher risk of cerebrovascular disease, HIV-infected women aged 25–29 years have a relatively higher risk of ischemic stroke (127), possibly due to HIV-related changes in the production of female endogenous sex hormones, including estrogen deficiency (127, 128). In addition, epigenetics may be involved in brain structural damage.

**Figure 3:**
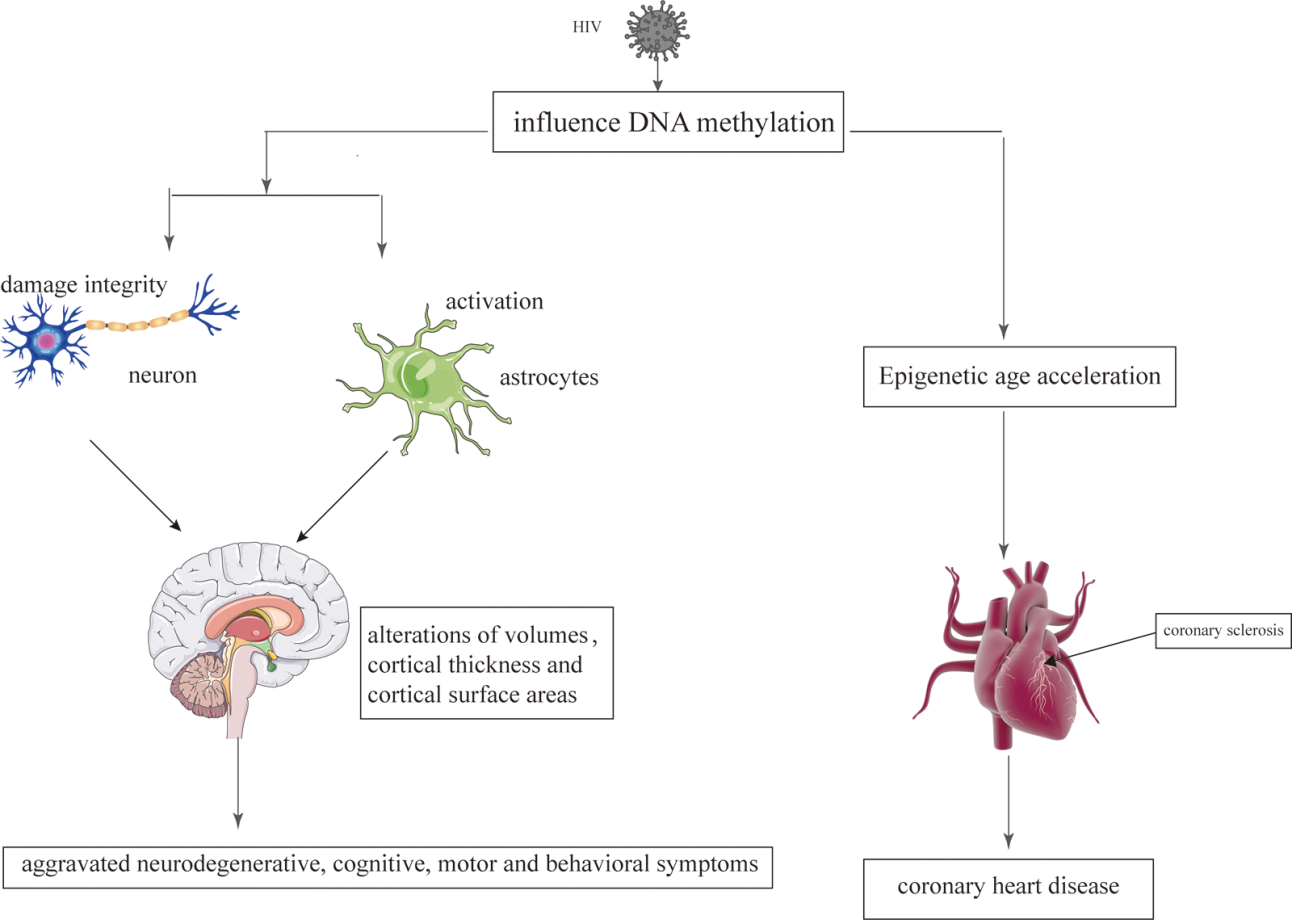
The epigenetic mechanisms modified by HIV on the brain and heart. HIV damages the brain and heart primarily by affecting DNA methylation.

Epigenetic mechanisms play an important role during the infection with retroviruses, including HIV which mediate the integration of the virus into the host genom (129). The change of epigenetic may be related to regional alterations of brain volumes, cortical thickness, cortical surface areas and neuronal microstructure (130). Persistence of latent HIV infection in the CNS was associated with increased levels of chromatin modifiers, which might result in abnormal transcriptomes, leading to inflammation, neurodegeneration, and neurocognitive impairment (131). Paula Desplats et.al detected changes in the expression of DNMT1, at mRNA and protein levels, that resulted in the increase of global DNA methylation. Moreover, Genome-wide profiling of DNA methylation showed differential methylation on genes related to neurodegeneration; dopamine metabolism and transport; and oxidative phosphorylation (132). Long-term HIV-Tat expression led to poorer short-and long-term memory, lower locomotor activity and impaired coordination and balance ability, increased astrocyte activation and compromised neuronal integrity, and decreased global genomic DNA methylation (133). N-terminal acetylation changes induced by viral infection might play a critical role in virus-host interactions in HIV infection (134). The epigenetic targets that might aid in understanding the aggravated neurodegenerative, cognitive, motor and behavioral symptoms observed in persons living with HIV and addictions Figure 3.

## 3. Indirect cardiovascular effects of HIV: effects of HIV on brain-heart regulation

The effects of HIV on the CNS encompass both metabolic dysfunction and structural damage. However, it is important to note that these two pathological states have distinct impacts on CS, both in terms of pathogenesis and clinical characteristics Table 2.

**Table 2:** Indirect cardiovascular effects of HIV.

Brain area	Neurocardiovascular function	Change of cardiovascular function caused by HIV
Medial prefrontal cortex	Regulate MAP and HR (164)	Longer QT interval (165)
Insular	1. Left insula:activating sympathetic (171) 2. Right insula: activating parasympathetic (172)	Uncertainties
Amygdaloid nucleus	Influence BP and HR (175–178)	Changes in BP and HR (182, 183)
Cingulate cortex	Hypertension and hypotensive (165, 189)	Disorder of HR and BP (186–188)
Hypothalamic	Regulate BP and HR (190, 191)	Disorder of HR and BP (72, 205).
Brainstem	Regulate cardiovascular function (206)	Bradycardia (208, 209)

### 3.1. CS alterations induced by metabolic dysfunction of CNS

**Figure 4:**
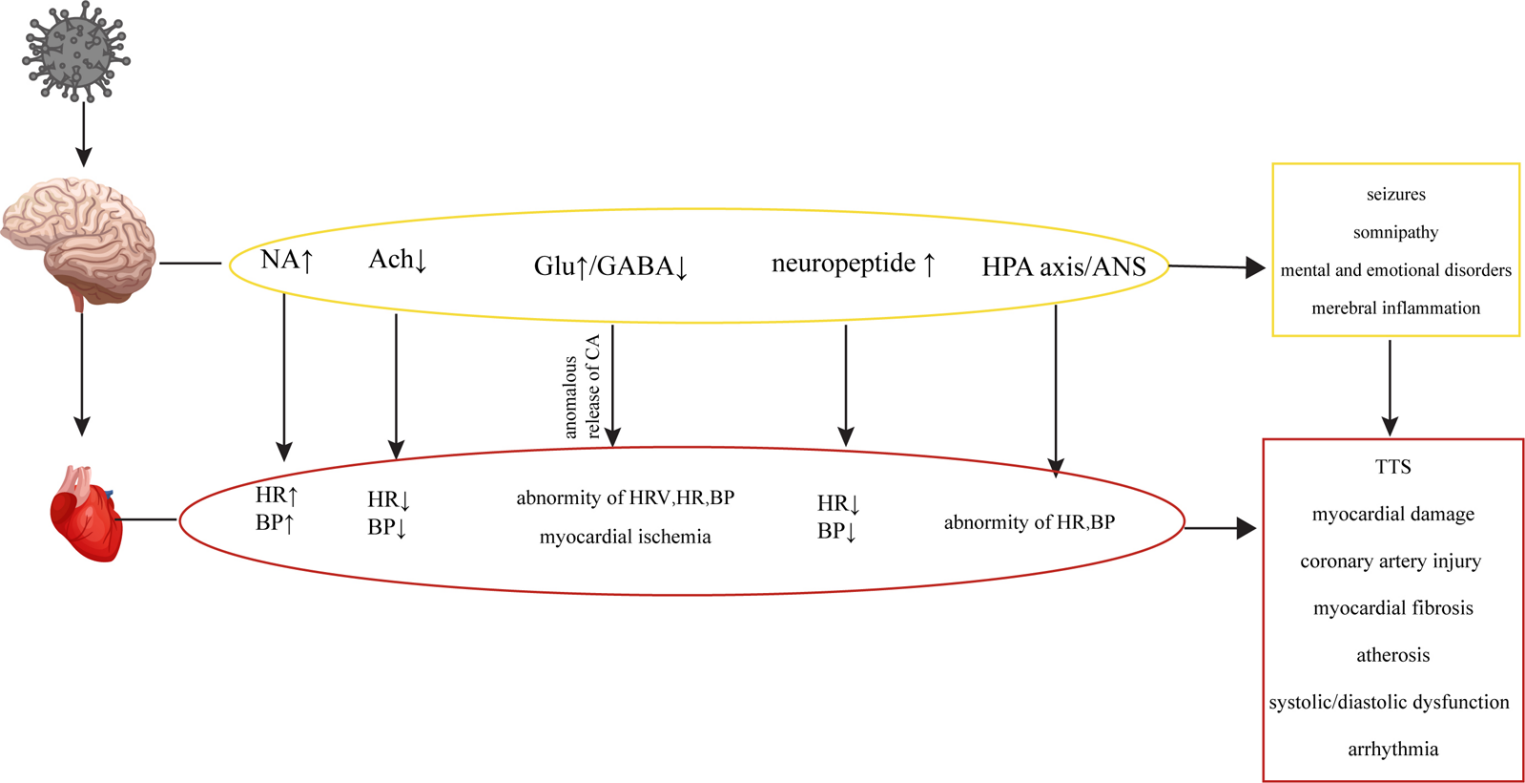
Cardiovascular diseases are caused by metabolic disorders of the CNS. HIV causes CNS metabolic disorders, leading to a series of changes in the cardiovascular system.

In the early stages of HIV infection, structural lesions in the brain may not be apparent. However, various metabolic disorders can occur, affecting neurotransmitters such as Noradrenaline (NA), Acetylcholine (Ach), catecholamines (CA), Glu, GABA and neuropeptides, whose abnormal levels may disrupt the regulatory network involving the brain, autonomic nervous system and CS, leading to cardiovascular abnormalities Figure 4 (17, 18, 88–90). While the concentration of NA in the peripheral blood of HIV patients may not show significant changes, there are notable alterations in the concentration of NA in the brain, which can contribute to abnormalities in cardiovascular activity (135, 136). The gp41 and gp120 proteins of HIV can stimulate the release of NA by regulating NMDA receptors in the brain, which activates the sympathetic nervous system, leading to tachycardia and elevated blood pressure (136–139). HIV-Tat protein can induce Ca^2+^-dependent Ach release through discrete amino acid sequences binding to different acceptance sites in the cortical cholinergic terminals, resulting in decreased heart rate and blood pressure (140, 141).

HIV infection can induce seizures by disrupting the balance between excitation and inhibition in the brain, known as an imbalance of excitation and inhibition (E-I imbalance), characterized by an increase in Glu levels and a decrease in GABA levels (109, 110, 142). The E-I imbalance in the brain can affect the normal regulatory mechanisms of the CS, leading to alterations in heart rate variability (HRV), changes in heart rate and blood pressure, and potentially causing transient myocardial ischemia (195). The E-I imbalance can also affect catecholamine secretion, leading to repetitive myocardial injury, chronic damage to the heart and coronary arteries, myocardial fibrosis, atherosclerosis, cardiac systolic and diastolic dysfunction, and arrhythmias within the CS (143, 144). In addition to its potential to induce epilepsy, the E-I imbalance in the brain can also impact the sleep patterns of HIV patients, contributing to abnormal functional and structural changes in the heart, leading to an increased risk of myocardial infarction (114, 145, 146). HIV patients frequently experience mental and emotional abnormalities due to the psychological stress associated with the disease itself and the effects of the virus on neurotransmitters in the brain (108, 147). Patients infected with HIV may experience Takotsubo syndrome (TTS) when subjected to both psychological and physical stress (148, 149). TTS is a notable condition highlighting the connection between mental stress, cortical activation, and cardiac disease. It is characterized by clinical symptoms resembling a myocardial infarction, accompanied by acute systolic apical left ventricular dysfunction triggered by physical or emotional stress (150, 151). TTS typically manifests with sudden onset chest pain, dyspnea and electrocardiogram changes similar to acute coronary syndrome, slightly elevated levels of myocardial enzymes, and transient abnormalities in ventricular wall movement unrelated to specific coronary perfusion area (152). Previous studies have demonstrated that HIV can cause dysfunction of the hypothalamic-pituitary-adrenal (HPA) axis and ANS, leading to abnormal levels of peripheral adrenaline and impaired regulation of the CS (135, 153, 154). Additionally, the levels of neuropeptides, which can directly impact the CS by increasing blood pressure and inhibiting sympathetic activity through their actions on the hypothalamus, have been found elevated in the brain (155–157). HIV could cause oxidative stress in the CNS and eventually result in abnormal baroreceptor and chemoreceptor signals transmitted by cardiac afferent fibers to the solitary tract nucleus, paraventricular nucleus and rostral ventrolateral medulla (RVLM), leading to sympathetic dysfunction, fatal arrhythmia, heart failure and myocardial ischemia (158–161).

### 3.2. CS injury in brain structural damage

**Figure 5:**
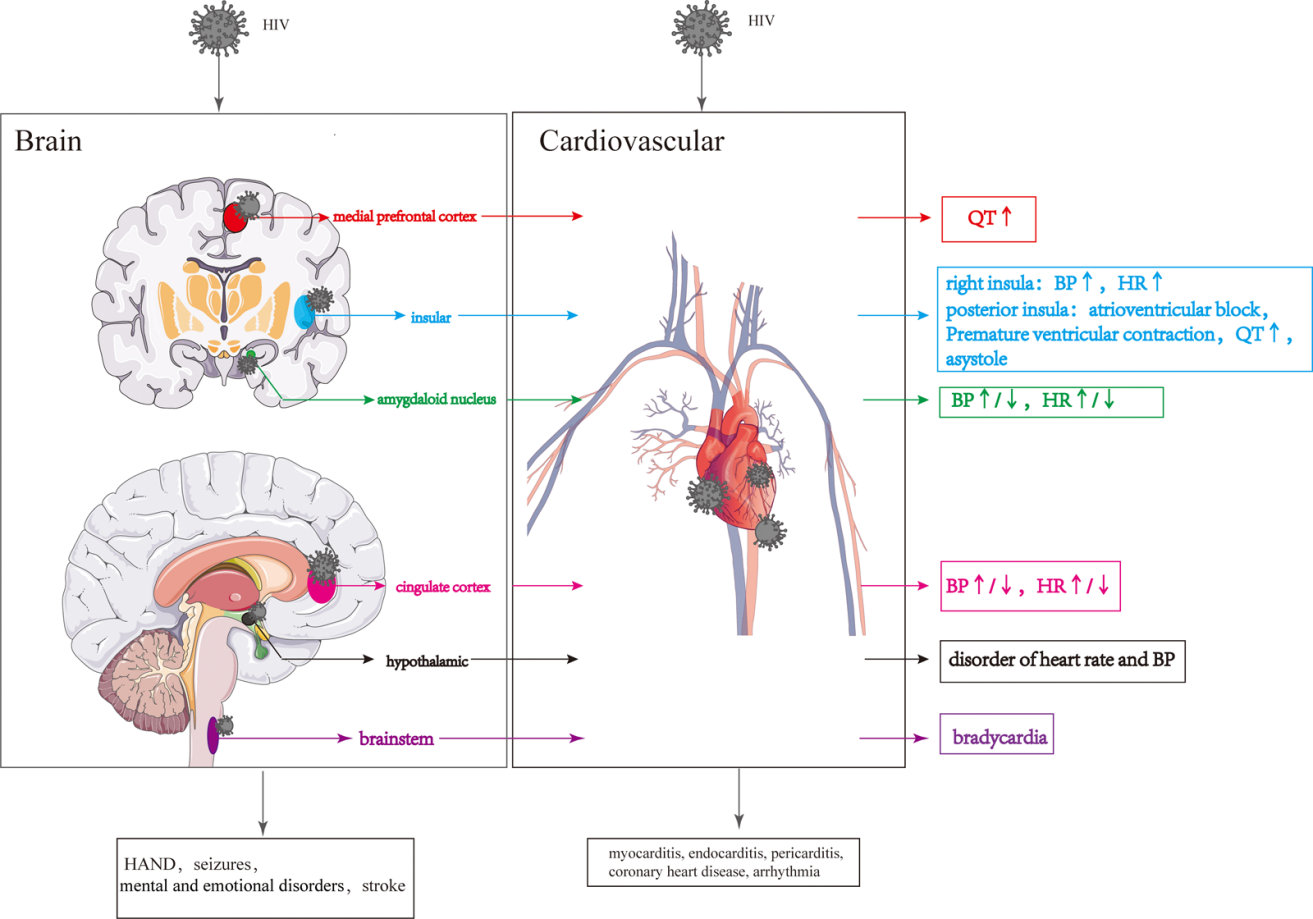
Cardiovascular lesions are caused by structural CNS damage and those directly caused by HIV. HIV invasion of the CNS causes changes in the different brain regions, resulting in changes in the cardiovascular system. HIV directly invades the cardiovascular system leading to various cardiovascular diseases.

Structural damage to the brain resulting from HIV infection can have more severe consequences, and the specific regions affected can influence the extent of cardiovascular damage. In particular, injury to the prefrontal cortical-insula-amygdala-cingulate cortical-hypothalamic-brain stem network is more likely to cause CS impairments Figure 5.

#### 3.2.1. Prefrontal cortex

The prefrontal cortex (PFC) can be divided into two regions based on functional, morphological, and evolutionary differences: the lateral PFC (LPFC) and the medial PFC (MPFC) (162). The ventral portion of medial prefrontal cortex (vMPFC), a portion of the MPFC, plays a crucial role in regulating the CS (163). Upon stimulation, the vMPFC activates the sympathetic nerve, increasing mean arterial pressure (MAP) (164). Notably, it has been reported that lower CD4 levels in HIV patients are associated with greater connectivity between the right vMPFC and right posterior insula and longer QT interval (165).

#### 3.2.2. Insular

The insula is involved in various physiological functions, including visceral sensation and visceral movement (166–168). The posterior insula receives and integrates signals related to visceral sensations, which are then projected to the anterior insula (169). After receiving visceral sensory signals from the posterior insular lobe, the anterior insular lobe sends out visceral movement signals, regulating visceral movement, including the CS (170). In HIV-infected individuals, damage to the gray matter of the right insular lobe can result in parasympathetic inhibition and sympathetic excitation, leading to increased heart rate and blood pressure (125, 171, 172). Overstimulation of the posterior insula can contribute to the development of conditions such as atrioventricular block, premature ventricular contractions, prolonged QT interval, and even asystole (173). The insula consists of two different types of neurons simultaneously: 27 percent responsible for sympathetic excitation and 9 percent responsible for sympathetic inhibition (174). Furthermore, the left and right insula have distinct roles in regulating the CS, with the left insula influencing sympathetic activity by regulating peripheral blood and cardiac adrenaline levels, while the right insula exerts the opposite effect by activating the parasympathetic nervous system (171, 172). Due to the insula's complex and multifaceted physiological functions, CS abnormalities resulting from insula damage can be subject to various uncertainties.

#### 3.2.3. Amygdaloid nucleus

The cortical nuclei, including the basolateral nucleus, cortical-like nuclei and central-medial nuclei, are situated in the medial temporal lobe (175). The amygdaloid nucleus, particularly the basolateral nucleus, receives and integrates signals from the cortex, brainstem and thalamus to regulate cardiovascular activity. These signals are then transmitted to the basal ganglia and subsequently reach the central part of the central medial nucleus (CeA). The CeA sends signals to the brainstem, hypothalamus, and other areas to influence blood pressure and heart rate (175–178). Glu secreted by the CeA increases heart rate and blood pressure by activating the sympathetic nervous system, while GABA secreted by the CeA reduces heart rate and blood pressure by activating the parasympathetic nervous system (179–181). In HIV-infected individuals, degeneration or volume loss in the amygdala may impair the transmission of cardiovascular regulatory signals to areas such as the brainstem and hypothalamus, leading to changes in blood pressure and heart rate (182, 183).

#### 3.2.4. Cingulate cortex

The cingulate cortex plays a role in receiving and integrating visceral sensory information, including changes in blood pressure, and it outputs signals that regulate autonomic, neuroendocrine, and cardiovascular responses (184). The posterior cingulate cortex can be further divided into the anterior cingulate cortex (ACC) and posterior cingulate cortex (PCC) (185). Activation of the ACC has been shown to reduce vagal tone and increase heart rate (186, 187), while inactivation of the ACC enhances the vagal tone and increases heart rate variability (188). HIV may cause impairment of ACC, which could change the heart rate and blood pressure. However, cardiovascular lesions caused by HIV injury to the cingulate gyrus are very complicated due to the presence of both the hypertension and hypotensive regions in the cingulate gyrus (165, 189).

#### 3.2.5. Hypothalamic

The hypothalamus, acting as a central hub for autonomic nerve outflow and the hypothalamic-pituitary-adrenal (HPA) axis, plays a vital role in regulating cardiovascular functions (190, 191). The hypothalamic paraventricular nucleus (PVN) releases corticotropin-releasing hormone (CRH), which then activates the HPA axis, leading to the release of adrenocorticotropic hormone (ACTH) from the pituitary gland. ACTH, in turn, stimulates the release of glucocorticoids from the adrenal gland, resulting in an increase in blood pressure (192). The activity level of CRH neurons located in the hypothalamic PVN can influence changes in blood pressure (193). Increased activity and/or number of CRH neurons can enhance CRH synthesis, transport and release, ultimately leading to elevated blood pressure (194). CRH neurons in the PVN receive catecholamine signals from brain regions such as the locus coeruleus (LC), nucleus of the solitary tract (NTS) and parabrachial nucleus (195–198), following which CRH neurons are activated, leading to an increased concentration of NA in the PVN, causing hypertension and an elevated heart rate (199, 200). Blood pressure regulation is also influenced by oxytocin (OXY) and arginine vasopressin (AVP), which are released from the supraoptic nucleus (SON), paraventricular nucleus (PVN) and suprachiasmatic nucleus (SCN) of the hypothalamus (201). Pro-oxytocin and pro-vasopressin, synthesized in the SON, PVN, and SCN, are transported, stored in the posterior pituitary, and released into the peripheral circulation to regulate the CS (202). In addition, OXY and AVP signals can project to various brain regions, including the olfactory bulb, orbitofrontal cortex, cingulate gyrus, amygdala, striatum, hippocampus, bed nucleus terminalis, suprachiasmatic nucleus, and autonomic ganglia (203). HIV-gp120 can invade the hypothalamus and directly stimulate the hypothalamic axis to induce cortisol secretion (204). HIV-Vpr and HIV-Tat can enhance glucocorticoid action by increasing the sensitivity of target tissues to glucocorticoids and stimulate the inflammatory cytokine HPA activity, resulting in cortisol secretion (205). In addition, Furthermore, HIV infection may reduce levels of oxytocin, which can disrupt the regulation of blood pressure (72).

#### 3.2.6. Brainstem

The brainstem consists of the midbrain, pons, and medulla oblongata, with the medulla oblongata particularly involved in cardiovascular regulation (206). When blood pressure increases, the nucleus tractus solitarius receives and integrates signals from baroreceptors, and then transmits these signals to neurons in the lateral caudal ventrolateral medulla (CVLM) (207), providing inhibitory signals to neurons in the rostral ventrolateral medulla (RVLM) (207). Subsequently, the inhibitory neural signals from RVLM neurons reduce the output of sympathetic signals from neurons located in the intermediolateral columns of cells in the spinal cord to peripheral organs, ultimately inhibiting cardiovascular activity (207). Furthermore, cardiac parasympathetic fibers originating from the dorsal vagus and hypochondriac nuclei of the medulla oblongata play a role in inhibiting cardiovascular activity. Brailoiu et al. suggested that HIV-Tat can stimulate the vagus nucleus, increasing parasympathetic activity in the heart and resulting in persistent bradycardia (208). Nagamitsu et al. also noted that structural changes in the medulla oblongata might occur in the later stages of HIV infection, which could potentially impact cardiovascular activity (209).

## 4. Direct effects of HIV on the CS

In AIDS patients, HIV infection is a significant contributor to the development of acquired heart disease, particularly symptomatic heart failure (210). Cardiovascular complications associated with HIV infection, such as subclinical electrocardiogram (ECG) changes, cardiomyopathy, and sudden cardiac death, are often observed in the later stages of AIDS (211). A model based on the AIDS Treatment Evaluation Cohort in the Netherlands revealed that 78% of HIV-infected individuals receiving ART were still diagnosed with cardiovascular disease, despite living longer (212). HIV infection increases the risk of various cardiac abnormalities, including those affecting the heart muscle, pericardium, heart valves, arterial vessels, and conduction system Table 3 (213).

**Table 3:** Cardiovascular diseases caused by HIV.

Cardiovascular diseases caused by HIV	Pathogenesis
Myocarditis	1. Direct invasion of myocardial (215, 216) 2. Directly induce myocardial inflammation (217)
Endocarditis	Staphylococcus aureus (229).
Pericarditis	Opportunistic infections (232)
Coronary heart disease	1. Infiltration of inflammatory cells and the obstruction of blood vessels (235) 2. Epigenetic changes (238)
Arrhythmia	Electrophysiological recombination of the heart (244)

### 4.1. Damage of HIV on the heart muscle

HIV-positive individuals are prone to myocarditis, with specific myocarditis accounting for approximately 50% of cases (214). The invasion of cardiomyocytes by HIV is a critical factor in developing specific myocarditis (215). Shaboodien et al. identified histological evidence of myocarditis in 44% of HIV patients using myocardial biopsy, suggesting that HIV can directly induce changes in myocardial inflammation (216). In addition, HIV can indirectly damage heart muscle cells, contributing to cardiac complications. Monsuez et al. demonstrated that HIV induces abnormal inflammation by infecting cardiac dendritic cells and endothelial cells, which mediate chronic inflammation by stimulating the production of tumor necrosis factor-α, interleukin-1 and omega-6, as well as other pro-inflammatory cytokines, leading to myocardial damage and dysfunction (217). HIV-induced cardiomyopathy occurs through mechanisms involving the invasion of cardiomyocytes by the virus and the apoptotic signaling triggered by HIV proteins like gp120, Tat and cytokines (218). Cheryl et al. showed that cardiomyocytes undergo apoptosis through both mitochondrion- and death receptor-controlled apoptotic pathways in HIV patients, which may be associated with gp120-induced apoptosis (219). Additionally, autoimmune mechanisms can play a significant role in myocarditis. HIV-associated B cells are stimulated to produce autoantibodies targeting the heart muscle, resulting in myocardial damage and systolic dysfunction (220).

The pathological features of HIV-associated cardiomyopathy are similar to those seen in patients without HIV infection, which primarily include ventricular dilation, a rounded apex, altered heart shape, and increased heart weight due to fibrosis and hypertrophy of cardiomyocytes (221, 222). Histologically, increased collagen in interstitial and endocardial fibers is a prominent characteristic of HIV-associated cardiomyopathy, along with myocardial cell hypertrophy, cardiomyocyte degeneration, myofibrillar loss, and intramuscular hydropic degeneration (221, 222). Before the widespread use of ART, symptomatic dilated cardiomyopathy with decreased ejection fraction was commonly observed in HIV-related cardiomyopathy. However, with the advent of ART, asymptomatic cardiomyopathy with or without abnormal ejection fraction has become more predominant (223, 224). Among asymptomatic HIV-infected patients, approximately 43.4% have diastolic dysfunction, while 8.3% have systolic dysfunction (225). The phenotype of HIV-associated dilated cardiomyopathy corresponds to the severity of immunosuppression, with a worse prognosis observed in patients with more severe immunosuppression (226). Patients with HIV-associated dilated cardiomyopathy may exhibit acute left heart failure symptoms (227).

### 4.2. Damage of HIV on the endocardium

In HIV-infected patients, the incidence of endocardial damage, often characterized by valve regurgitation, can be as high as 77% (228). Individuals with HIV infection who have a CD4 count of less than 50 cells/mm^3^ and a high viral load (greater than 100,000 copies/ml) are at a four-fold increased risk of developing infective endocarditis (IE), with staphylococcus aureus being a common pathogenic organism associated with this condition (229).

### 4.3. Damage of HIV on the pericardium

Before the widespread use of ART, the prevalence of pericardial effusion in HIV patients was reported to be as high as 25% (230) but has decreased since the widespread use of ART (231). HIV-associated pericardial effusion is often associated with opportunistic infections due to the patient's compromised immune function (232). In addition to opportunistic infections, pericardial effusion can also be caused by capillary leakage resulting from cytokine activation late in HIV infection (232, 233). Moreover, direct invasion of the pericarditis by HIV is also a direct cause of pericarditis (234).

### 4.4. Damage of HIV on artery

HIV-related arterial vessel injury is a distinct condition characterized by the infiltration of inflammatory cells and the obstruction of blood vessels, which can weaken the walls of the vessels and lead to the formation of aneurysms (235). Following HIV infection, viral replication, immune system disruption, and intestinal microbial translocation can trigger chronic systemic inflammation, leading to pathological manifestations such as dyslipidemia, thrombosis, chronic inflammation of vascular endothelial cells, and ultimately the development of conditions such as coronary heart disease(CHD) (236).

CHD is also considered to be an age-related heart disease (237). Compared to the individuals without HIV, HIV-infected patients have a higher risk of developing CHD, which tend to occur at an earlier stage (9). With regard to CHD that occurs at an earlier stage, Huang et al. believe that it is related to epigenetic changes caused by HIV (238). Epigenetic age acceleration is verified in HIV-infected patients, which may be related to HIV causing DNA methylation (238). DNA methylation is one of the most studied epigenetic markers, which involves changes in the DNA that are influenced by environmental factors (239). DNA methylation is the common HIV-induced epigenetic changes (240). Therefore, HIV interferes with the genome of the cells by affecting the methylation of the DNA, which could promote the aging process and increase the risk of CHD Figure 3 (241, 242).

### 4.5. Arrhythmia caused by HIV

ECG abnormalities commonly observed in HIV patients include ventricular conduction defects, such as isolated ST-T abnormalities and prolonged QT interval (243). Apart from the effects of medications, electrolyte imbalances and ANS dysregulation, arrhythmias in HIV-infected patients are also associated with the impact of HIV on the cardiac conduction system (244). *Nef*, the constituent gene of HIV, is also a major determinant of HIV pathogenicity, leading to the entire electrophysiological recombination of the heart (244). Judith Brouillette et al. suggested that *Nef* could lead to a 50% reduction in the outward potassium current of repolarization in ventricular myocytes, thereby prolonging the duration of ventricular action potential (245). The *Nef* genome can also result in a 30% reduction in depolarization of sodium current (246). HIV-Tat prolongs the QTc interval by increasing ROS production and decreasing hERG current in cardiomyocytes (247). In addition, the atrial fibrillation risk (AF) of HIV patients, which is related to the lower CD4 count or (and) and the higher viral load, is higher than that of the non-HIV population (248).

## 5. Overcome the effects of HIV on the brain-heart axis

The dysfunction of the CS is considered as a fatal disease in HIV-infected patients. Diagnosis and therapeutics for the damage of brain and heart are important methods and approaches to overcome the effects of HIV on the brain-heart axis. Through pharmacological and non-pharmacological interventions, as well as emerging technologies, researchers are making significant strides in improving outcomes for individuals affected by brain and heart-related conditions.

### 5.1. Diagnosis for brain damage

#### 5.1.1. Clinical manifestations

In the early stages of brain damage,the clinical manifestations are characterized by mild difficulties with concentration, motor symptoms and focal cortical deficits (92). As the disease advances, the clinical manifestations are characterized by significant motor dysfunctions, signs of frontal lobe release, cramps and hyper-reflexes, cognitive dysfunction, neuropathic pain, paresthesia, and abnormal gait (86, 120, 121, 249).

#### 5.1.2. Cerebrospinal fluid

Detection of viral load in cerebrospinal fluid (CSF) is an important means to assess whether HIV has invaded the CNS (250). However, viral load levels of HIV in CSF and the count of CD4+ T cells in blood are not necessarily positively correlated with the severity of brain damage (84–87, 251). Some biochemical substances(including glucose, ATP, lactate, Glu, GABA, NA, and catecholamine, et al.) and biomarkers(t-tau, *p*-tau, *β*-amiloid42, neopterin, and S100β,et al.) of CSF are utilized to assess metabolic dysfunction and Structural damage of CNS (104, 106–108, 136, 137, 143, 144, 252–255). In addition, integrated brain on a chip and automated organ-on-chips systems,as well as extracellular vesicles, may be employed to evaluate various neurological damage caused by HIV (256–258).

#### 5.1.3. Electroencephalograph

Electroencephalograph (EEG) is commonly utilized for assessing electrophysiological function in the brain. Tinuper et al. found that abnormal EEGs are correlated with CNS involvement and borderline EEGs may be correlated with asymptomatic patients (259). Barco et al.observed a statistically significant correlation between working memory test Trail Making B and delta waves in patients infected with HIV (255). For functional connectivity, connectivity in alpha, beta, theta, and delta band was decreased in the HIV/AIDS patients (255). Compared to the healthy people, the smaller late positive potential and larger P200 amplitudes were observed in HIV/AIDS patients (260).

#### 5.1.4. Magnetic resonance

Magnetic resonance (MRI), a widely used neuroimaging test, is considered to play an important role in the diagnosis of HAND (261). MRI can be employed to observe structural changes in the brain. In addition, some functional MRI (fMRI) tests, including susceptibility weighted imaging (SWI), diffusion tensor imaging (DTI), magnetic resonance spectroscopy (MRS), are utilized to verify the metabolic dysfunction and Structural damage of CNS caused by HIV, as well as CNS metabolic disorders (262–264).

### 5.2. Therapeutics for brain damage

ART has reduced the prevalence of HIV-associated dementia, but the persistent brain damage caused by HIV is an unmet challenge. Improving ART effectiveness in suppressing HIV replication in the CNS by either increasing penetration into the CNS is a strategy for protecting CNS. Reducing HIV replication in the central nervous system can inhibit the release of neurotoxins from activated microglia and astrocytes (265). As ART regimens are developed, the adjunctive neuroprotective therapies must accelerate. Anti-excitotoxicity, calcium channel blockers, antioxidants, and anti-inflammatory drugs are considered as adjunctive neuroprotective therapies for HIV-infected patients (265, 266). The neurotrophic factors are also applied to protect CNS (267).

### 5.3. Diagnosis and therapeutics for heart diseases

Diagnosis for HIV-associated heart diseases is similar to those observed in HIV-uninfected patients (223, 226, 230). EEG, ultrasonic cardiogram, coronal artery angiography, left ventriculography, myocardial enzyme, and markers of heart failure are often used to identify HIV-associated heart diseases (211, 268–270). Except for the need for ART, the therapeutics for HIV-associated heart diseases is similar to HIV-uninfected patients (225, 230, 236). Moreover, the nanomedicines which have been used in the treatment of cancer, may be applied to treat HIV associated heart diseases (271–273).

## 6. Limitations and future directions

Diseases of the cardiovascular system caused by the CNS are more complex and deadly (268). The clinicians and researchers get a new theoretical basis from the concept of the brain-heart axis (274, 275). They think about the role of the brain-heart axis in the face of diseases of the CNS and(or) cardiovascular system, such as stroke (276), seizures (277) and TTS (278). However, the limitations of this review should be acknowledged. No sufficient evidences about the effects of HIV on brain-heart axis were found. We only infer from the current study that HIV may have an impact on the brain-heart axis. There are two main future directions regarding the effects of HIV on the brain axis. On the one hand, a large amount of clinical data, especially CNS data in HIV-infected patients with cardiovascular disease, should be collected. Through these data, we could further understand CNS function in HIV-infected patients with cardiovascular disease. On the other hand, *in vitro* and *in vivo* models of HIV-infected CNS should be established to research the changes in the cardiovascular system.

## 7. Conclusion

In summary, the nervous system is crucial in regulating the CS through a complex neural network. However, HIV infection can cause functional and structural damage to the nervous system, thereby affecting the neural network's regulation of the CS and ultimately leading to abnormalities in the CS of patients, significantly impacting their quality of life. Thus, it is important to closely monitor changes in their nervous system and take a comprehensive approach to address the alterations in their CS when diagnosing and treating HIV patients.
